# Synergistic effects of *Malva sylvestris* gum and chitosan in bilayer films: Optimizing physical and functional properties with *Salvia abrotanoides* essential oil

**DOI:** 10.1016/j.fochx.2025.103113

**Published:** 2025-09-30

**Authors:** Zeinab Jaderi, Motahare Pirnia, Farideh Tabatabaei Yazdi, Seyyed Ali Mortazavi, Arash Koocheki

**Affiliations:** The Department of Food Science and Technology, Ferdowsi University of Mashhad, Iran

**Keywords:** Edible bilayer films, Active packaging, Barrier properties, *Malva sylvestris* gum

## Abstract

This study developed edible bilayer films from *Malva sylvestris* gum (MSG) and chitosan (CH), with a focus on incorporating *Salvia abrotanoides* essential oil (EO) as a bioactive additive. EO addition remarkably decreased moisture content from 0.096 ± 0.028 to 26.08 ± 0.15, solubility from 65.84 ± 0.54 to 31.45 ± 0.19, while thickness increased significantly from 0.096 ± 0.028 to 0.118 ± 0.09 for MSG/CH and MSG/CH-2 %EO, respectively. Color and optical characteristics were determined. SEM images revealed film's microstructure and FTIR analysis evaluated chemical interactions within polymer matrix. Differential scanning calorimetry revealed a reduction in T_g_, from 27 °C (MSG/CH) to 10.8 °C (MSG/CH-2 %EO), indicating enhanced chain mobility. Mechanical evaluation showed a decrease in tensile strength (12.5 to 5.1 MPa) but an improvement in elongation at break (12.2 % to 18.6 %) (*p* < 0.05). Antioxidant evaluation demonstrated a significant rise in DPPH scavenging activity at 2 % EO in bilayer films. These findings highlight EO-incorporated MSG/CH films as a promising biodegradable packaging material with enhanced functional properties.

## Highlights

• Malva sylvestris gum-chitosan (50:50) films showed balanced mechanical strength and barrier properties for packaging.
• The incorporation of *Salvia abrotanoides* EO improved thermal stability, antioxidant activity, and elongation at break while reducing moisture content and transparency.
• SEM and ATR-FTIR analyses confirmed strong hydrogen bonding and electrostatic interactions, representing compact and cohesive bilayer films.
• Differential scanning calorimetry revealed EO incorporation reduced Tg via plasticization and increased Tm through crystalline reinforcement.

## Graphical abstract

**Figure:**
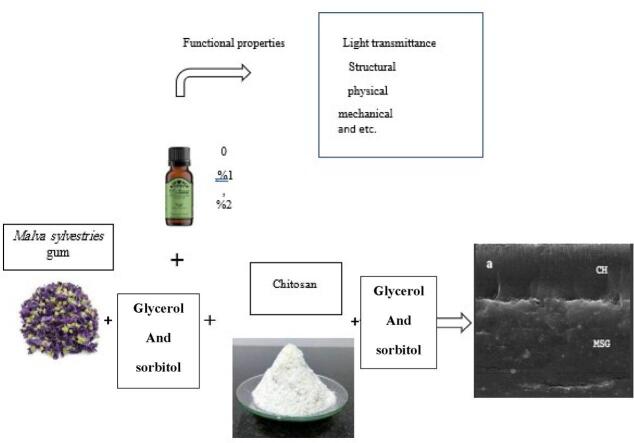

## 1 Introduction

The growing awareness of plastic pollution, resource depletion, and the environmental persistence of petroleum-derived packaging materials has intensified the search for sustainable alternatives. Conventional polymers such as polyethylene (PE) and polyethylene terephthalate (PET) dominate global food packaging markets but are neither biodegradable nor renewable, contributing significantly to landfill and marine microplastic accumulation. These challenges have fueled an urgent shift towards the development of biodegradable, bio-based films for food packaging, capable of offering both functionality and ecological safety (Heras et al., 2024; Liu et al., 2024).

In this context, biopolymer-based edible films derived from polysaccharides, proteins, or lipids have gained traction. Their compatibility with food systems, renewability, and potential to carry bioactive compounds make them attractive for modern packaging applications(Cao, Liu, et al., 2024; Cao, Wang, et al., 2024; Jahed et al., 2025). Among these, chitosan (CH), a natural cationic polymer derived from chitin, is widely recognized for its excellent film-forming properties, antimicrobial activity, and biodegradability. However, chitosan films often exhibit limitations such as brittleness and sensitivity to moisture, which compromise their mechanical integrity under humid conditions (Fatima et al., 2024; Liu et al., 2024). To overcome these challenges, composite film systems blending chitosan with other polysaccharides such as *Malva sylvestris* L. which is known as a medicinal plant traditionally used for its soothing, anti-inflammatory properties and rich in mucilage polysaccharides (Hamedi et al., 2015). The gum fraction (MSG) extracted from *M. sylvestris* demonstrates gelling, emulsifying, and thickening properties, along with antioxidant potential due to its phenolic constituents. Despite its promise, MSG has been underutilized in the design of functional edible films, and little is known about its interactions with chitosan in composite or bilayer systems (Jaderi et al., 2023). Bilayer edible films represent a novel direction in sustainable packaging, allowing the integration of materials with complementary hydrophilic, hydrophobic, and structural characteristics. This layered configuration not only enhances mechanical robustness and barrier efficiency but also enables controlled release of incorporated bioactive compounds (Pirnia et al., 2022).

Among functional bio additives used in food packaging, essential oils (EOs) are particularly valued for their broad-spectrum antimicrobial and antioxidant properties(Burt, 2004; Sharma et al., 2021). However, their inherent volatility and sensitivity to environmental factors often necessitate incorporation into a biopolymeric matrix that can stabilize and modulate their release.

*Salvia abrotanoides* essential oil (EO) is a bioactive extract from a *Lamiaceae* species which is native to the Middle East and South Asia and known for its high concentrations of phenolic monoterpenes and flavonoids with proven efficacy against foodborne pathogens (Jaderi et al., 2022). Despite its therapeutic potential, *Salvia abrotanoides* EO has not yet been explored in combination with *Malva sylvestris* gum (MSG) and chitosan (CH) in composite edible films.

While chitosan-based bilayers have been previously studied with various natural gums, the pairing of MSG and CH remains unreported, particularly in formulations containing functional plant-derived EOs. Therefore, this study introduces an innovative MSG-CH bilayer film system, reinforced with *Salvia abrotanoides* EO to monitor the synergistic attributes of both polymers and the bioactivity of EO compounds.

## 2 Materials and methods

### 2.1 Materials

*Malva sylvestris* flowers were harvested from Kalat Naderi, Razavi Khorasan province, Iran. Chitosan (CH) with a deacetylation degree >95 % and viscosity of 200–400 mPa.s was obtained from Tamadkala Co., Tehran, Iran. Analytical-grade glycerol, sorbitol, and Tween 80 were sourced from Sigma-Aldrich (USA) and used as plasticizers and emulsifying agents. Folin-Ciocalteu reagent was acquired from Sigma Chemical Co., and ethyl alcohol was obtained from Tamadkala Co., Iran. *S. abrotanoides* essential oil (EO) was prepared following Jaderi et al. (2022) with purity of 90 %. Additional reagents, including sodium chloride, anhydrous calcium chloride, DPPH, and gallic acid, were purchased from Merck (Germany).

### 2.2 Extraction of polysaccharides

The genus and species of *M. sylvestris* were authenticated by the Plant Research Institute of Ferdowsi University of Mashhad, with herbarium identification number E-1236. Fresh flowers were cleaned, washed with distilled water, and dried at 35 °C. The dried material was ground (Moulinex 320P, France) and sieved to 0.5 mm particle size, then stored at 4 °C in airtight containers. To eliminate impurities, 100 g of grounded *M. sylvestris* was treated with 80 % ethanol at a ratio of 1:8 (*w*/w) and heated in a 60 °C water bath. After centrifugation, the residue was rinsed with acetone and re-dried at 40 °C. The process was repeated three times to maximize purity. The resulting polysaccharide was dissolved in distilled water at 65 °C, centrifuged at 7000 rpm, and precipitated with 90 % ethanol. The gum was then dried, powdered, and stored under dry, cool conditions(Pirnia et al., 2022; Yekta et al., 2020).

### 2.3 Zeta potential

Aqueous MSG dispersions were prepared at a concentration of 1.5 mg/mL and pH 7. Zeta potential measurements were conducted using a dynamic light scattering (DLS) de*v*ice (Malvern Instruments, UK). Samples were diluted with deionized water, loaded into disposable cells, and analyzed to determine the electrostatic stability of the MSG (Zhang et al., 2021).

### 2.4 Film elaboration

#### 2.4.1 Preparation of chitosan (CH) films

For CH film production, 15 mg of chitosan was dissol*v*ed in 1 % (*v*/v) acetic acid at 55 °C with constant stirring. Glycerol was added at 50 % (*w*/w) of chitosan, followed by heating at 40 °C for 1 h. The solution was degassed by centrifugation and poured onto Petri dishes, which were then dried at 35 °C. Films were conditioned at 25 °C and 53 % relati*v*e humidity (RH) before analysis(Pirnia et al., 2022).

#### 2.4.2 Preparation of *M. sylvestris* gum (MSG) films

*Malva sylvestris* gum (MSG) was dissolved in distilled water at concentrations of 0.75 %, 1 %, and 1.5 % (*w*/*v*). Plasticizers including glycerol and sorbitol in a 1:1 ratio were added at 40 %, 50 %, and 60 % (w/w) based on the gum content. *S. abrotanoides* essential oil (EO) was incorporated at concentrations of 1 % and 2 % (*v*/v), along with 2 % (v/v) Tween 80 as a surfactant. The resulting emulsion was homogenized at 7000 rpm for 5 min using an Ultra-Turrax homogenizer. Film-forming solutions were then cooled to 4 °C, degassed, and cast into Petri dishes for drying at 35 °C and 58 % relative humidity over 24–48 h(Han et al., 2018; Jaderi et al., 2022).

#### 2.4.3 Formation of bilayer films

Bilayer films were prepared by adding a chitosan layer over pre-dried MSG films, following the two-step casting technique as described by (Pirnia et al., 2022), with some modifications to meet the study requirements.

### 2.5 Evaluation of film properties

#### 2.5.1 Thickness measurement

Film thickness was measured with a digital micrometer (QLR digital-IP54, China) at five randomly selected points on each film. The average of these measurements was calculated as per described by (Zareie et al., 2020).

#### 2.5.2 Moisture content (MC)

Moisture content was determined gravimetrically by drying film samples at 105 °C for 24 h (Zareie et al., 2020). Measurements were repeated in triplicate, and MC was calculated using the following formula:

$$M_{t}=\left(\frac{w_{i}-w_{d}}{w_{i}}\right)\times 100$$

M_t_: MC of the sample (%); W_i_: Initial sample weight in grams(g); W_d_: Dried sample weight in grams (g).

#### 2.5.3 Solubility in water (S_w_)

Water solubility was assessed based on modified protocol by (Choo et al., 2024). Briefly conditioning films at 75 % RH for 48 h, then immersing 3 × 3 cm^2^ samples in distilled water with agitation for 8 h at room temperature. Samples were oven-dried at 110 °C to constant weight, with solubility (%) calculated as:

$$\text{Water solubility}\;\left(\%\right)=\frac{\text{initial}\;\mathrm{dry}\;\text{weight}-\text{final}\;\mathrm{dry}\;\text{weight}}{\text{initial}\;\mathrm{dry}\;\text{weight}}\times 100$$

#### 2.5.4 Water vapor permeability (WVP)

WVP was determined using a gravimetric method based on ASTM E96–95. Film samples were affixed to permeation cups containing anhydrous calcium chloride and stored in a desiccator at 75 % RH. Weight changes were recorded at 12-h intervals over 3 days until stable (Choo et al., 2021). WVP was calculated as:

$$\text{WVTR}=\frac{S}{A}$$

$$\mathrm{WVP}=\frac{\text{WVTR}\times X}{∆p}$$

WVTR: Water vapor transmission rate; S: Slope of the regression model; A: Permeation area (m^2^); X: Film thickness (mm); ∆P: Water vapor pressure difference across the film (kPa).

#### 2.5.5 Mechanical properties

Tensile strength (TS) and elongation at break (EB) were evaluated using a texture analyzer (TAXT-plus, Stable Micro Systems). Films (2 × 5 cm) were clamped, and tests were conducted with an initial grip separation of 30 mm and cross-head speed of 1 mm/s. Results were averaged from triplicates (Pirnia et al., 2022).

$$\text{Tensile strength}\;\left(\mathrm{MPa}\right)=\frac{\text{Fmax}}{W\times T}$$

$$\text{Elongation}\;\mathrm{at}\;\text{break}\left(\%\right)=\frac{L-L0}{A}\times 100$$

F_max_: Maximum force applied during the testing; W: Width of the film (mm); T: Thickness of the film (m); L: Final length of the film after testing (mm); L_0_: Initial length of the film before testing (mm); A: Distance between the blades or grips of the testing device.

#### 2.5.6 Thermal properties

Differential scanning calorimetry (DSC) was performed using a NETZSCH DSC 214 Polyma instrument. Approximately 3 mg of each film sample was sealed in standard aluminum pans and heated from −100 °C to 200 °C at a rate of 5 °C/min under a nitrogen atmosphere. The glass transition temperature (Tg) and melting point (Tm) were recorded using the instrument software (Ghanbarzadeh et al., 2010).

#### 2.5.7 Transparency

Transparency and opacity were measured using an Ultrascan VIS spectrophotometer (HunterLab, Germany). Films (5 × 50 mm) were analyzed for transmittance in the 200–800 nm range based on the method provided by (Khan et al., 2025). Transparency was calculated as:

Transparency (A/mm) = −log T/x.

A = Absorbance at each wavelength; T = Transmittance (%) at each wavelength; x = Film thickness (mm).

#### 2.5.8 Color measurement

Color parameters (L*, a*, b*) were measured using a HunterLab colorimeter, with standard calibration (Ghanbarzadeh et al., 2010). Color difference (∆E) were calculated using the following equation:

$$∆E=\sqrt{{\left(L-L_{0}\right)}^{2}+{\left(a-a_{0}\right)}^{2}+{\left(b-b_{0}\right)}^{2}}$$

where L_0_, a_0_, and b_0_   are the color values of the control film, and L, a, and b are those of the treated samples.

#### 2.5.9 Scanning Electron microscopy (SEM)

Cross-sectional analysis of cryo-fractured films was conducted using SEM (LEO 1450 VP, Germany). Samples were coated with 20 nm gold for conductivity, with SEM images captured at 20 kV (Pirnia et al., 2022).

#### 2.5.10 ATR-FTIR spectroscopy

ATR-FTIR spectroscopy (Thermo Nicolet AVATAR 370) was used to analyze film structure in the range of 400–4000 cm^−1^, with each spectrum averaged over 16 scans at 4 cm^−1^ resolution (Akhter et al., 2019).

#### 2.5.11 Antioxidant activity by DPPH assay

Antioxidant activity was assessed using the DPPH radical scavenging method. Films (25 mg) were dissolved in distilled water, mixed with DPPH solution, and incubated in the dark for 60 min. Absorbance at 517 nm was measured (Siripatrawan & Harte, 2010). Radical scavenging activity (%) was calculated as:

$$\text{Radical scavenging activity}\;\left(\%\right)=\left(\frac{A_{\text{control}}-A_{\text{sample}}}{A_{\text{control}}}\right)\times 100$$

A_control_: Absorbance value of control; A_sample_: Absorbance value of sample.

### 2.6 Statistical analysis

The data from the experiment were presented as mean ± standard deviation. To compare the samples, a one-way analysis of variance (ANOVA) was conducted, followed by Duncan's multiple-range test. A significance level of *p* < 0.05 was chosen to indicate statistical significance. The statistical analysis was carried out using SPSS 19.0 software (SPSS Inc., Chicago, IL, USA).

## 3 Results and discussion

1.1. Selection of Optimal MSG/CH Formulation for Antimicrobial Bilayer Film Preparation

The 50:50 ratio of *Malva sylvestris* gum (MSG) to chitosan (CH) was identified as the optimal formulation for developing bilayer antimicrobial films due to its balanced properties considering all properties. While the 70:30 MSG/CH ratio exhibited high water vapor permeability (WVP) with satisfactory physical attributes, and the 30:70 ratio showed improved barrier properties but compromised mechanical strength, the 50:50 ratio offered an ideal compromise. This balanced formulation provided moderate WVP, strong mechanical stability, and flexibility, aligning well with recent findings on the benefits of polysaccharide-protein matrices in food packaging applications. Consequently, the 50:50 MSG/CH ratio was selected for further composition with *S. abrotanoides* EO (Table 1).

1.2. Film thickness, moisture content, water solubility, and water vapor permeability

**Table 1:** Bilayer films formulation.

Film	MSG solution (%)	CH solution (%)
MSG70/CH30	70	30
MSG50/CH50	50	50
MSG30/CH70	30	70

The result of evaluation of different film's physicochemical properties is shown in Table 2. Based on the presented data, film thickness was significantly influenced by both the film composition and the incorporation of EO. The MSG monolayer films showed the lowest thickness values, while the addition of a chitosan (CH) layer in the bilayer structure led to a significant increase in thickness value (*P* < 0.05). Incorporation of EO into MSG monolayer films resulted in an increasing trend in thickness, with the 2 % EO formulation exhibiting the highest value among all treatments (*P* < 0.05). This trend can be attributed to the presence of hydrophobic oil droplets within the polymer matrix, which can disrupt intermolecular interactions between polysaccharide chains and hinder tight packing during drying, leading to a bulkier microstructure (Ayazi et al., 2024). (Cai et al., 2020; Zhou et al., 2021) likewise reported similar trends.

**Table 2:** Physical properties of monolayer and bilayer films containing 1 % and 2 % EO.

Film Type	Thickness (mm)	Water Solubility (%)	Moisture Content (%)	WVP (×10^−11^ g/m·s·Pa)
MSG	0.080 ± 0.014^a^	77.20 ± 0.66^a^	29.43 ± 0.17^a^	3.2 ± 0.03^d^
MSG/CH	0.096 ± 0.028^b^	65.84 ± 0.54^b^	27.66 ± 0.63^bc^	6.4 ± 0.056^d^
MSG −1 % EO	0.127 ± 0.049^c^	58.89 ± 0.59^c^	29.32 ± 0.13^b^	3.3 ± 0.12^cd^
MSG - 2 % EO	0.141 ± 0.021^e^	44.38 ± 1.19^d^	28.96 ± 0.04^ab^	3.8 ± 0.31^b^
MSG/CH -1 % EO	0.104 ± 0.077^bc^	39.31 ± 0.16^e^	26.88 ± 0.02^cd^	6.6 ± 0.31^ab^
MSG/CH - 2 % EO	0.118 ± 0.09^d^	31.45 ± 0.19^f^	26.08 ± 0.15^d^	6.8 ± 0.04^ab^
CH	0.095 ± 0.028^b^	19.44 ± 0.28^g^	22.35 ± 0.63^e^	8.5 ± 0.25^a^

a The data are reported as the average value with the corresponding standard deviation.

b Significant differences among groups within the same column are denoted by distinct superscript letters. (*P* < 0.05).

In MSG/CH bilayer films, the addition of EO produced a remarkable increase in thickness compared to the control bilayer sample and these differences were statistically significant (*P* < 0.05). The compact and cohesive nature of the CH layer may have restricted the extent of swelling and oil migration during the film-forming process. CH monolayer films showed thickness values similar to the bilayer without oil, consistent with previous reports describing the dense structure of chitosan-based films (Stefanowska et al., 2024).

The water solubility (WS) of the films was notably influenced by both the film formulation and the incorporation of EO (Table 2). Among the control samples, MSG monolayer films exhibited the highest WS, reflecting the highly hydrophilic nature of polysaccharides and the loose molecular network of MSG, which allows rapid penetration and dissolution in aqueous media. Incorporating a chitosan (CH) layer in the bilayer films markedly reduced WS (*P* < 0.05), consistent with the lower solubility and more compact structure of chitosan-based matrices reported previously (Stefanowska et al., 2024).

The incorporation of EO significantly decreased WS in both monolayer and bilayer films (*p* < 0.05). This reduction can be attributed to the hydrophobic character of the oil, which creates a more water-resistant network by reducing polymer-water interactions and increasing matrix hydrophobicity. Similar effects have been observed in starch- and pectin-based films incorporated with essential oils, where the oil droplets act as a moisture barrier and hinder water penetration (Pirouzifard et al., 2019; Zhou et al., 2021). In MSG monolayer films, increasing EO concentration from 1 % to 2 % further decreased WS, suggesting improved dispersion and interaction of oil droplets within the polymer at higher concentration. (Zhou et al., 2021) also reported increasing the interaction of essential oil with the film's hydroxyl groups, leading to a reduced number of free hydroxyl groups in the matrix and shrinking the WS value. In bilayer structures, the combination of CH and EO produced the lowest WS values among all treatments, indicating a synergistic effect of the inherently low-solubility CH layer and the oil's hydrophobic domains. This trend aligns with findings by(Syafiq et al., 2021), who reported enhanced water resistance in biopolymer films when hydrophobic compounds were integrated into multilayer configurations.

The moisture content (MC) of the films was significantly affected by both the film composition and the incorporation of EO (Table 2). MSG monolayer films exhibited the highest MC values among all formulations, reflecting the strong hydrophilic nature of MSG polysaccharides and their high water-binding capacity. The addition of a CH layer in the bilayer structure markedly reduced MC (*P* < 0.05), which can be attributed to the denser, less hydrophilic network of chitosan that limits water retention within the film matrix. Incorporation of EO into MSG monolayer films resulted in a reduction in MC, with the most evident decrease observed at 2 % EO (P < 0.05). This trend is consistent with previous reports, where hydrophobic essential oils reduced the number of free hydrophilic sites available for water sorption, thereby lowering the film's equilibrium moisture level (Syafiq et al., 2021; Zhou et al., 2021).

Similarly, EO addition to MSG/CH bilayer films further decreased MC compared to their control bilayer representatives (*P* < 0.05). This effect likely arises from the synergistic barrier function of the compact CH layer and the hydrophobic EO domains, which together limit water-polymer interactions and restrict water uptake. Such reductions in MC due to EO incorporation have been reported in cassava starch-EO films (Zhou et al., 2021) and potato starch films with sage EO (Pirouzifard et al., 2021), which stated the oils created a more water-resistant network.

As shown in Table 2, the WVP of the films varied significantly depending on both the film composition and the incorporation of EO (*P* < 0.05). The lowest WVP values were observed for MSG (3.2 × 10^−11^ g/m·s·Pa) and MSG-1 % EO (3.3 × 10^−11^ g/m·s·Pa) films, indicating excellent water vapor barrier performance. Incorporation of CH into the MSG matrix increased WVP, suggesting that the bilayer structure may have increased moisture diffusion. Among all treatments, CH monolayer films exhibited the highest WVP (8.5 × 10^−11^ g/m·s·Pa), consistent with the more hydrophilic nature of chitosan and its relatively open network structure, which facilitates water vapor migration. In general, EO incorporation into MSG monolayers did significantly impair barrier properties, whereas in MSG/CH bilayers, EO addition resulted in higher WVP, possibly due to partial disruption of the compact CH layer by dispersed oil droplets during film formation (*P* < 0.05). Similar reports were published indicated similar results (Zhou et al., 2021). Zhou et al. (2021) found that incorporating cinnamon essential oil (CEO) into cassava starch films increased WVP at 2.5 % CEO compared to the control. The authors attributed this intensification to the disruption of the starch polymer network by dispersed oil droplets, which created more permeable pathways for water vapor spreading. Future studies should also evaluate the films' oxygen permeability, as it plays a critical role in determining their function for food packaging application when they comes in contact to foods with sensitivity to oxygen.

1.3. Color and transparency measurement

The addition of *S. abrotanoides* EO had a noticeable impact on the color and optical properties of both monolayer and bilayer films, as summarized in Table 3. As the EO concentration increased, remarkable changes were observed in the L*, a*, and b* indicators and in the total color difference (ΔE). The lightness index (L*), which represents the brightness of the films, significantly decreased with EO incorporation (P < 0.05). For instance, the L* value of the MSG monolayer film without EO was 73.76, which declined to 69.21 and 65.17 upon addition of 1 % and 2 % EO, respectively. A similar trend was observed in bilayer MSG/CH films, where the L* index dropped significantly from 75.79 to 68.66 as EO concentration increased (P < 0.05). This reduction in lightness likely results from the presence of high levels of phenolic compounds in *S. abrotanoides* EO, which are known to absorb light, especially in the lower visible spectrum, thereby darkening the film and reducing its transparency. It also may be associated with Millard reactions during film solution production (Jaderi et al., 2023; Pirnia et al., 2022). Likewise, (Ayazi et al., 2024) stated that films with a higher pea protein isolate (PPI) concentration also exhibited increased redness and yellowness, potentially due to Maillard reactions between amino groups and carbohydrates.

**Table 3:** Color of mono- and bilayer films containing *S. abratanoides* EO.

Film	L^⁎^	a^⁎^	b^⁎^	∆E	Transmittance(%)
MSG	73.76 ± 0.15^c^	−1.49 ± 0.07^c^	27.95 ± 0.84^c^	33.39 ± 2.72^c^	70.57 ± 0.25^a^
MSG/CH	75.79 ± 0.26^b^	−2.06 ± 0.77^d^	22.02 ± 0.10^e^	36.87 ± 2.01^b^	72.34 ± 0.11^b^
MSG-%1 EO	69.21 ± 0.14^e^	−1.06 ± 0.84^b^	28.89 ± 0.21^b^	39.97 ± 3.71^a^	70.68 ± 0.03^a^
MSG-%2 EO	65.17 ± 0.19^f^	−0.77 ± 0.35^b^	29.44 ± 0.14^a^	27.40 ± 1.76^d^	71.04 ± 0.021^ab^
MSG/CH-%1 EO	71.26 ± 1.49^d^	−1.4 ± 0.14^c^	24.96 ± 0.14^d^	32.56 ± 6.78^c^	73.12 ± 0.08^bc^
MSG/CH-%2 EO	68.66 ± 0.77^e^	−1.15 ± 0.07^b^	28.52 ± 0.43^b^	36.94 ± 6.62^b^	73.92 ± 0.18^bc^
CH	83.10 ± 0.19^a^	−2.64 ± 0.02^e^	16.29 ± 0.84^f^	18.50 ± 0.83^e^	74.70 ± 0.21^c^

a The data are reported as the average value with the corresponding standard deviation.

b Significant differences among groups within the same column are denoted by distinct superscript letters. (*P* < 0.05).

Furthermore, the a* index increased with EO concentration, and changes were consistent across both film types and aligned with the yellowish hue observed during visual inspection of the samples. The total color difference (ΔE) further quantified color changes, confirming that EO incorporation induces noticeable and statistically significant color modifications. These findings are in line with previous reports.

For example, (Ayazi et al., 2024) reported that the color properties of the films were significantly influenced by the increment of essential oils concentration and the ratio of biopolymers. Specifically, increasing the cumin essential oil (CEO) concentration generally cause an increase in the a* index (redness/greenness), b* index (yellowness/blueness), and total color differences. This increase in b* was particularly attributed to the pigments naturally present in CEO, and possibly sage seed gum (SG) as well, contributing to a yellowish or brownish tint. Similarly, (Zhou et al., 2021) demonstrates that the inclusion of essential oils significantly affect the color characteristics of films, often resulting in a noticeable yellowish hue and an increase in total color difference.

The transmittance values presented in Table 3 offer further insight into the visual and colorimetric variations caused by the incorporation of *S. abrotanoides* EO into both monolayer and bilayer film matrices. Generally, the addition of EO influenced the optical clarity of the films, though not in a linear trend. For instance, MSG films without any EO showed 70.57 % of transparency. This percentage rised slightly to 70.68 % when 1 % EO was added, and then to 71.04 % with 2 % EO. This small increase might be due to minor changes in the film's structure or how well the EO spread out, but overall, the difference was not statistically different (*P* > 0.05).

These findings are similar to what other studies have shown. For example, Ayazi et al. (2024) found that adding cumin essential oil made the films less transparent, especially at higher concentrations. Similarly, Zhou et al. (2021) observed that cassava starch films with cinnamon EO were less clear than films without EO.

In control bilayer film transparency increased significantly (*p* < 0.05), however incorporation of EO didn't leave significant impact on transparency (*p* > 0.05).

Even though our MSG/CH films containing EO showed only small increases in transmittance, this might suggest specific interactions between MSG and chitosan that allow the EO to spread more evenly. This indicates that adding EO only slightly affects the optical properties while keeping the films clear enough for use in food packaging.

1.4. Thermal properties

The thermal properties of the films were investigated using differential scanning calorimetry (DSC), and the resulting thermograms are presented in Fig. 6. As DSC profile indicates, the first thermal event was identified as the glass transition temperature (T_g_), while the second peak corresponded to the melting temperature (T_m_), both of which are closely associated with the crystalline structure of the polymeric films (Peesan et al., 2005). Below T_g_, the films stay in a rigid, glassy state with slight molecular mobility. Once the temperature goes beyond T_g_, the films exhibit enhanced softness and flexibility due to an increase in molecular motion increases and intermolecular distances (Ghasemlou et al., 2011).

For the control samples, the monolayer MSG film and the MSG/CH bilayer film without EO displayed T_g_ values of 36 °C and 27 °C, respectively. Incorporation of S. *abrotanoides* EO caused a decrease in T_g_, reflecting the plasticizing effect of the oil counterpart. Specifically, T_g_ values declined to 10 °C and 8.4 °C for monolayer films containing 1 % and 2 % EO, and to 23 °C and 10.8 °C for bilayer films at the same concentrations. This reduction can be attributed to the hydrophobic compounds within the EO, which weaken polymer-polymer interactions, enhance chain mobility, and thereby increase the flexibility of the films. Similar reductions in T_g_ due to essential oil incorporation have been documented in alginate- and starch-based matrices (Ojagh et al., 2010; Zhou et al., 2021).

Conversely, the addition of EO increased the T_m_ values of the films, suggesting that the hydrophobic interactions of the oil constituents enhanced the stability of crystalline domains despite the increase in overall flexibility. For the MSG monolayer films, T_m_ values rose from 66 °C (control) to 68 °C and 79 °C for 1 % and 2 % EO, respectively. The MSG/CH bilayer films also showed an increase. This upward trend in T_m_ may be explained by the molecular weight and hydrophobicity of EO compounds, which facilitate more ordered packing and stabilization of crystalline regions. Comparable results were reported by Ojagh et al. (2010), who observed that clove EO enhanced the thermal stability of chitosan films, while Zhou et al. (2021) demonstrated similar improvements in the heat resistance of polysaccharide-protein composite films upon EO incorporation.

1.5. Mechanical properties

The tensile strength (TS) and elongation at break (EAB) values of the films are presented in Fig. 7a and Fig. 7b, respectively. Both parameters were significantly (*P* < 0.05) affected by the polymer composition and the incorporation of *S. abrotanoides* EO. Control MSG films exhibited the highest TS (∼24 MPa), which can be attributed to the dense hydrogen bonding within the MSG matrix, creating a rigid and cohesive network. In contrast, MSG/CH-EO2 % monolayer films showed the lowest TS (∼7 MPa) which is consistent with previous reports, where the incorporation of cinnamon essential oil into starch-gelatin films markedly reduced TS due to the presence of oil droplets that interfered with the continuous polymer matrix (Syafiq et al., 2020). The MSG/CH bilayer films without EO (∼12 MPa) indicate intermediate strength, suggesting that while some degree of interfacial bonding occurs between the layers, structural discontinuities at the interface limit stress transfer efficiency. These results are aligned with trends reported in Cassava starch films with cinnamon essential oil, where TS decreased from 2.08 MPa to 0.80 MPa with increasing EO content (Zhou et al., 2021). This weakening effect is attributed to EO molecules disrupting polymer–polymer hydrogen bonding and introducing hydrophobic domains that interfere with network continuity. Comparable TS reductions have also been reported in research conducted by (Syafiq et al., 2020).

On the other hand, Elongation at break (EAB) values (Fig. 6b) increased significantly (*p* < 0.05) upon EO incorporation. The MSG films showed low flecibility (∼8 %), while CH monolayers exhibited higher extensibility (∼18 %). Addition of *S. abrotanoides* EO enhanced flexibility across all formulations (*p* < 0.05), with MSG-EO2 % reaching ∼15 % and MSG/CH-EO2 % reaching around the CH monolayer at ∼18 %. Essential oil incorporation modifies the polymeric network by weakening intermolecular bonding, thereby increasing molecular mobility and improving film flexibility. Comparable increases in elongation have been reported for cassava starch films, where EAB improved from 3.52 % to 42.17 % at 2 % oil addition (Zhou et al., 2021)

1.6. Zeta potential

The zeta potential of *Malva sylvestris* gum (MSG) solutions offer critical insights into their electrostatic behavior and suitability for film development. As depicted in Fig. 2, the MSG solution exhibited a zeta potential centered around −52 mV, signifying a strong negative surface charge and robust colloidal stability. This inherent stability effectively minimizes molecular aggregation, thereby facilitating the creation of uniform films which is a particularly vital characteristic when MSG is integrated with positively charged polymers such as chitosan (CH). The pronounced negative surface charge of MSG significantly enhances its capacity to form electrostatic interactions with CH, leading to the development of well-integrated bilayer films with improved mechanical and barrier properties. Choma et al. (2025) highlights how chitosan acts as an effective carrier for essential oil components, leading to enhanced biological properties. The bioactive compounds present in *Salvia* species, such as phenolic compounds and flavonoids, are known for their antioxidant and antimicrobial properties, making *Salvia abrotanoides* essential oil a promising candidate for such applications (Woźniak et al., 2024).

These observations are corroborated by research from Wang et al. (2023), who demonstrated that nanofillers, specifically chitin nanofibers, establish potent electrostatic and hydrogen bonding interactions within chitosan matrices. Their work confirmed that these interactions contribute to enhanced film compactness, thermal stability, and tensile properties, with both zeta potential and FTIR analyses validating the formation of stable film networks(Wang et al., 2023). Similarly, Hua et al. (2021) showed that nanoparticles incorporated into CH-based films improved zeta potential, which facilitated the formation of stable emulsions with essential oils, ultimately resulting in films with superior barrier and mechanical attributes (Hua et al., 2021). These compelling examples collectively underscore the principle that a well-defined and robust zeta potential profile, such as the −52 mV observed for MSG, directly contributes to successful biopolymer integration and subsequent enhancements in overall film quality.

Furthermore, the electrostatic compatibility between MSG and CH, as elucidated through zeta potential profiling, not only supports the formation of homogeneous films but also promotes the effective integration of bioactive agents like *Salvia abrotanoides* EO. This balanced electrostatic environment likely aids in the uniform distribution and sustained retention of EO within the film matrix, which in turn significantly reinforces the antioxidant and antimicrobial performance of films which are two crucial factors for advanced food packaging applications.

1.7. ATR-FTIR

The ATR-FTIR spectra presented in Fig. 3 provide a detailed understanding of the interactions between *Malva sylvestris* gum (MSG) and chitosan (CH) in the MSG/CH bilayer films, compared to their individual films. This analysis aims to elucidate the electrostatic interactions and hydrogen bonding within the bilayer structure, which are crucial for enhancing film stability and functionality, especially in food packaging applications.

As shown in both panels of Fig. 3, moderate absorbance peaks are observed in the 3200–3500 cm^−1^ range, attributed to the -OH stretching vibrations. The spectra demonstrate a significant shift towards lower wavelength in the bilayer films, with the broad O—H stretching peak shifting from 3240 cm^−1^ in the standalone CH film and 3194 cm^−1^ in the MSG film. This shift to lower wavenumbers suggests increased hydrogen bonding between MSG and CH layers within the bilayer configuration. Li et al. (2017) reported similar shifts, linking them to enhanced hydrogen bonding, which plays a vital role in creating stable biopolymer networks. This increase in hydrogen bonding likely contributes to the cohesive integrity of the MSG/CH bilayer, enhancing its water resistance and mechanical properties (Chen et al., 2021; Zhang et al., 2022).

In addition, the spectra reveal significant changes in the regions associated with amide and carboxyl groups, representing electrostatic interactions. Specifically, the characteristic carboxyl group peak of MSG around 1610 cm^−1^ and the amide I and II peaks of CH at 1638 cm^−1^ and 1569 cm^−1^ exhibit noticeable shifts in the bilayer film. In the bilayer configuration, the amide I and II peaks shift to 1625 cm^−1^ and 1549 cm^−1^, while the MSG carboxyl peak shifts to 1598 cm^−1^. These shifts towards higher wavenumbers in the bilayer film indicate electrostatic interactions between the negatively charged carboxyl groups of MSG and the positively charged amine groups of CH. Such electrostatic interactions enhance film homogeneity and structural stability, consistent with findings by Chen et al. (2019) and Li et al. (2020), who observed similar interactions in other polysaccharide-chitosan bilayer systems.

Li et al. (2020) further highlighted that these electrostatic interactions increase the number of hydrogen bonds between the amine groups of CH and carboxyl groups of other polysaccharides, reinforcing the film structure. These findings align with the observed shifts in the MSG/CH bilayer films, suggesting that both hydrogen bonding and electrostatic interactions are at play, creating a robust network within the film matrix. Such structural integration between MSG and CH enhances the bilayer's mechanical strength, thermal stability, and barrier properties, as previously discussed.

These ATR-FTIR spectral results validate the choice of a 50:50 MSG/CH ratio, as it balances the interactions between MSG and CH, forming a stable and cohesive bilayer. The enhanced structural and functional properties of the MSG/CH bilayer films make them highly suitable for sustainable food packaging applications. Additionally, this stable matrix supports the incorporation of bioactive agents, such as *Salvia* essential oil (EO), further extending the film's antimicrobial efficacy and functionality in preserving food products.

In conclusion, the ATR-FTIR analysis confirms that the combination of MSG and CH in a bilayer structure promotes substantial hydrogen bonding and electrostatic interactions, enhancing the film's durability and performance. These interactions provide a solid foundation for developing versatile and eco-friendly packaging solutions with added antimicrobial properties, meeting both environmental and functional demands in modern food packaging.

### 3.1 SEM observation

**Fig. 1:**
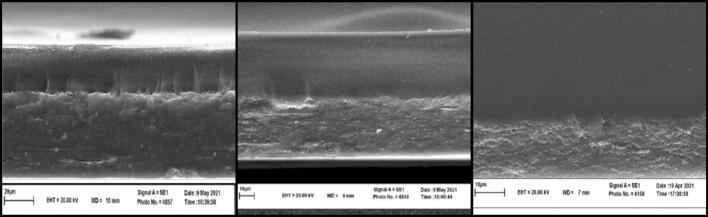
SEM images of fracture cross section for different *Malva Sylvestris* gum (MSG) and chitosan (CH) bilayer films. MSG:CH ratio is respectively a) 70:30 b) 50:50 c) 30:70.

Fig. 1 represents the morphological features of the MSG:CH bilayer films which were characterized by Scanning Electron Microscopy (SEM) to investigate structural organization and adhesion between layers. Fractured cross-sections of films prepared at different ratios of 70:30, 50:50, and 30:70 show obvious differences in compactness, porosity, and the integration between the interfaces, an important factor affecting the mechanical and functional properties of the films. The 70:30 film (MSG:CH) exhibits a perfect layer separation between MSG and CH layers, which is also reflected in the SEM image shown in Fig. 1a. The upper chitosan layer is smooth and compact, whereas the lower layer of MSG has a rather rougher and porous texture.

**Fig. 2:**
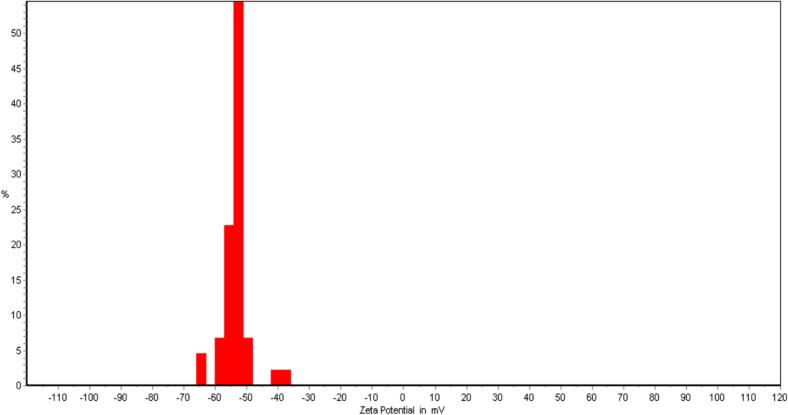
Zeta potential of polysaccharide solution of *Malva sylvestris.*

**Fig. 3:**
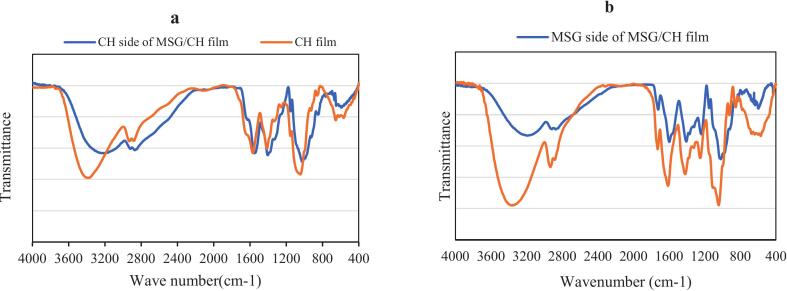
ATR-FTIR spectra of CH film with MSG/CH bilayer film (a) and MSG film with MSG/CH bilayer film (b). Abbreviation: *Malva Sylvestris* gum (MSG), Chitosan (CH).

**Fig. 4:**
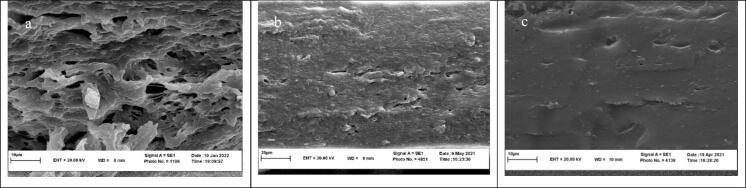
SEM images of cross-section of films. (a: MSG-2 % EO; b: MSG-1 %EO; and c: MSG).

**Fig. 5:**
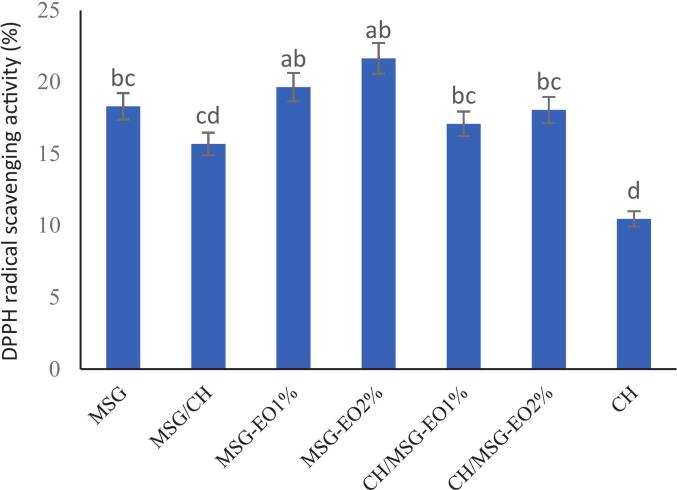
DPPH radical scavenging activity.

**Fig. 6:**
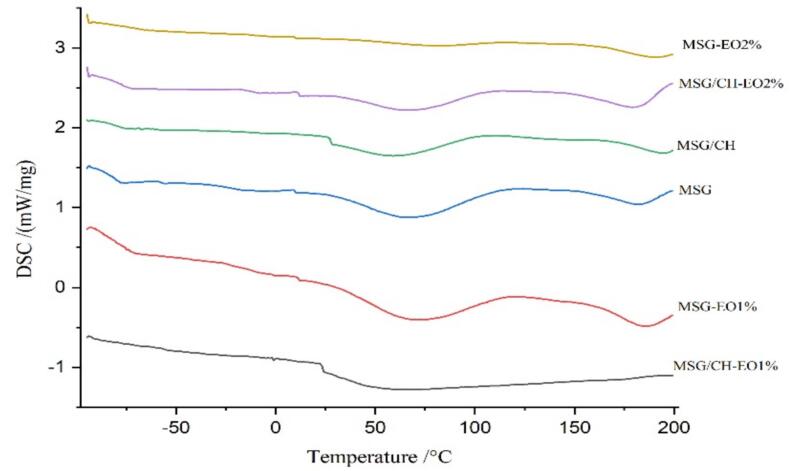
Thermogram of edible films containing different concentrations of EO.

**Fig. 7:**
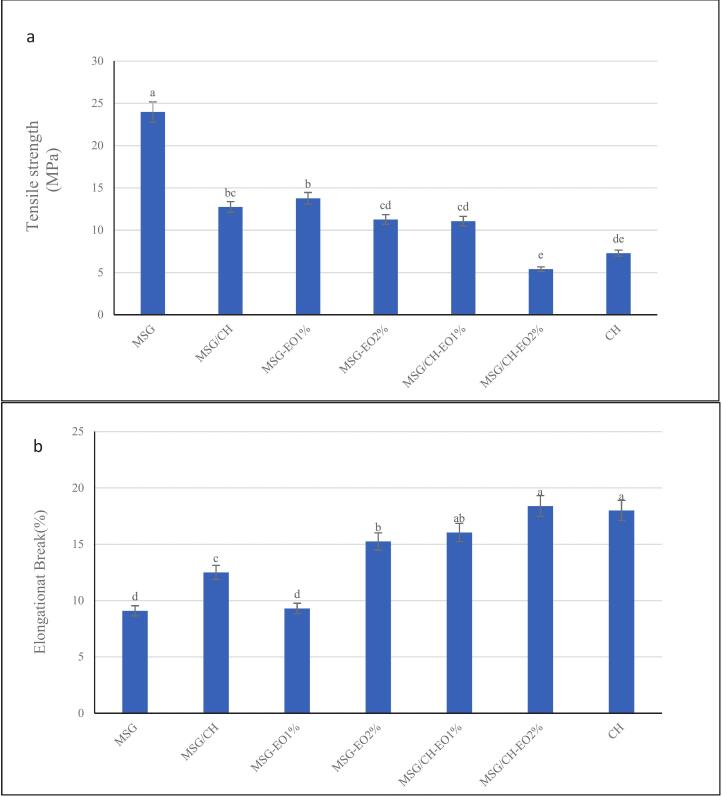
Mechanical properties of different films. Different letters show significant differences among film formulations (one-way ANOVA, *P* ≤ 0.05). Data are presented as means ± standard deviation.

Presence of cracks in the MSG layer implies brittleness, perhaps due to the weaker intermolecular forces than chitosan. Such behavior may indicate that the films with higher composition of MSG, while flexible, will readily undergo mechanical failure when some stress is applied. The SEM of the bilayer film 50:50 (MSG:CH) (Fig. 1b) demonstrates an enhanced adhesion between both layers. This would also mean better compatibility and therefore better miscibility of the polymers, as evidenced by the smoother interface. In this composition, porosity in the MSG layer is less than that obtained in film 70:30 and thus represents a better homogeneous structure. Such a balanced weight ratio could balance the properties of flexibility and mechanical resistance and be suitable for biodegradable packaging or any other application which requires both the properties, like intermediate barrier films. While by opposite, it could be observed in the SEM image for 30:70 MSG-CH that the bilayer film exhibited a compact and homogenous structure of high integrity dominated by the chitosan layer, whose interface with the materials turned out to be just very hardly noticeable, hence showing huge integration and good compatibility.

The smooth, continuous morphology, with a minimum of pores and cracks, suggests higher mechanical strength and durability. Such characteristics make the composition ideal for applications that require structural integrity, such as antimicrobial coatings, wound dressings, or protective layers in packaging materials. In general, the trend obtained by SEM analysis indicates an increase in compactness and adhesion of the chitosan content in bilayer films. This is explained by the hydrogen-bond-forming and strong intermolecular interactions of chitosan that improve structural integrity and decrease porosity. While the reverse occurs for high content of MSG, which introduces flexibility but at the expense of structural weaknesses due to a less compact arrangement and generally brittle nature.

These findings therefore underscore that optimization of the MSG:CH ratio shall be performed in view of specific application requirements where the flexibility and strength should be optimized for desired performance. The structural differences pointed out in this work underline the importance of composition-dependent morphology for functional properties of bilayer films. This can be done by varying the ratio of MSG:CH-, from flexible, porous structures that could be used for coatings to rigid compact layers suitable for applications in protection. These insights set a basis for the elaboration of biopolymeric materials with improved performance in various fields such as packaging, biomedical, and environmental applications.

Fig. 4 presents scanning electron microscopy (SEM) images of the cross-sections of different films, revealing distinct structural characteristics that reflect the interactions and compatibility between *Malva sylvestris* gum (MSG) and chitosan (CH) in the bilayer formulation. The micrographs highlight variations in film morphology across different compositions, which are crucial for understanding the mechanical integrity and barrier properties of each film type.

In Fig. 4a, the cross-section of the standalone MSG film shows a highly porous and irregular structure. This porous morphology indicates limited cohesive strength within the MSG matrix, likely due to insufficient intermolecular interactions and weak hydrogen bonding. The presence of these open pores could lead to increased water vapor permeability (WVP) and decreased mechanical strength, making it less suitable for direct food packaging applications that require strong barrier properties. The structural weaknesses in the MSG film also reflect the need for blending with other polymers, such as chitosan, to improve its functionality.

Fig. 4b displays the cross-section of the standalone CH film, which exhibits a relatively dense and layered structure compared to the MSG film. Natural biopolymer characteristics associated with chitosan contributes to a more cohesive and continuous morphology, with fewer visible pores. This compact structure is a result of stronger intermolecular interactions within chitosan, particularly through hydrogen bonding. The denser configuration of the CH film likely provides enhanced mechanical strength and a reduced rate of water permeability, making it more suitable for applications where barrier properties are prioritized. However, despite its cohesive structure, standalone chitosan films can be brittle, requiring blending with flexible biopolymers like MSG to enhance flexibility and handling properties.

Fig. 4c presents the cross-section of the MSG/CH bilayer film, which was optimized at a 50:50 ratio. This bilayer film shows a more uniform and compact structure with minimal porosity, indicating successful integration between the MSG and CH layers. The smooth and cohesive morphology suggests strong hydrogen bonding and electrostatic interactions at the MSG-CH interface, as confirmed by the ATR-FTIR analysis discussed earlier. This cohesive structure reduces porosity, which likely enhances the film's barrier properties and mechanical stability, while providing flexibility due to the balanced composition of MSG and CH. The minimized porosity also suggests improved water resistance, supporting the application of MSG/CH bilayer films in food packaging where moisture barrier properties are crucial.

These SEM observations confirm the complementary nature of MSG and CH in forming a bilayer film with optimal structural properties. The 50:50 MSG/CH formulation results in a compact, smooth, and low-porosity structure, highlighting its potential as a sustainable and functional composition for food packaging. This enhanced morphology is also expected to support the effective incorporation of *Salvia* essential oil (EO) for more antimicrobial functionality, aligning with the goals of creating bioactive and environmentally friendly packaging solutions.

### 3.2 Antioxidant activity

As shown in Fig. 5, the DPPH radical scavenging activity of various film formulations is presented, providing insights into the antioxidant properties of *Malva sylvestris* gum (MSG), chitosan (CH), and the impact of incorporating *Salvia abrotanoides* essential oil (EO). A DPPH scavenging assay was employed to evaluate the antioxidant activity of the films, and the results reveal a statistically significant enhancement (*P* < 0.05) in DPPH scavenging activity at higher EO concentrations. The MSG film shows moderate DPPH radical scavenging activity (∼18 %), which can be attributed to the natural antioxidant compounds in *M. sylvestris* gum. However, the antioxidant activity decreases slightly in the MSG/CH bilayer without EO, possibly due to the dilution effect of CH, which has limited inherent antioxidant properties. On the other hand, the incorporation of EO at 1 % and 2 % concentrations substantially increases the antioxidant activity. The MSG-EO2 % and CH/MSG-EO2 % films exhibit the highest DPPH scavenging activities, with values reaching approximately 21.65 % and 19.37 %, respectively. This increase is consistent with findings by our previous research by Jaderi et al. (2022), which attribute the antioxidant capacity of *S. abrotanoides* EO to its rich phenolic content, specifically camphor, α-pinene, and 1,8-cineole. These compounds are known for their strong free radical scavenging abilities, enhancing the films' potential to protect packaged food from oxidative spoilage. The higher concentration of EO (2 %) in the MSG and MSG/CH films caused the most pronounced increase in antioxidant activity, further supporting the role of phenolic compounds in EO as key contributors to radical scavenging.

Similar results were reported by (Ayazi et al., 2024) who claimed that the antioxidant potential significantly increased (P < 0.05) by the improvement in CEO concentration, as raising the concentration of cumin essential oil (CEO) from 1 % to 2 % led to a pronounced improvement in radical scavenging activity. This enhancement is closely linked to the higher levels of phenolic constituents contributed by CEO, which act as efficient hydrogen donors and readily neutralize free radicals. Also, (Syafiq et al., 2021) observed that the antioxidant potential of films was significantly enhanced (P < 0.05) with the addition of cinnamon essential oil (CEO). Specifically, they noted a dose-dependent improvement in radical scavenging activity when CEO concentration was raised from 1 % to 2 %. This effect was attributed to the enrichment of phenolic compounds present in CEO, which serve as efficient hydrogen donors capable of neutralizing free radicals . taken together results suggests that MSG/CH films containing EO can serve as active food packaging materials, providing promising antioxidant benefits that help to extend shelf life by minimizing oxidative damage.

## 4 Conclusion

This study developed and characterized edible bilayer films composed of *Malva sylvestris* gum and chitosan, highlighting their potential as sustainable and functional materials for food packaging. The optimized 50:50 MSG/CH formulation indicated a desirable physical, mechanical, and barrier properties, ensuring both integrity and flexibility. Incorporation of EO further improved barrier properties, antioxidant activity, and elongation at break, confirming its role as an effective functional additive. Overall, these results suggest that MSG/CH bilayer films incorporated with EO represent a promising biodegradable and bioactive packaging strategy with potential to extend food shelf life and can be further applied on food models in future research.

## CRediT authorship contribution statement

**Zeinab Jaderi:** Writing – original draft, Visualization, Software, Methodology, Investigation, Formal analysis, Data curation. **Motahare Pirnia:** Writing – review & editing, Writing – original draft, Software, Methodology, Conceptualization. **Farideh Tabatabee Yazdi:** Resources, Project administration, Methodology, Funding acquisition, Conceptualization. **Seyyed Ali Mortazavi:** Supervision, Methodology, Conceptualization. **Arash Koocheki:** Validation, Resources, Project administration.

## Declaration of competing interest

The authors declare that they have no known competing financial interests or personal relationships that could have appeared to influence the work reported in this paper.

## Data Availability

Data will be made available on request.
